# When does Orthodontics make a difference? Threshold effects in attractiveness perception and hiring intentions

**DOI:** 10.1590/2177-6709.31.2.e2625314.oar

**Published:** 2026-05-18

**Authors:** Gil Guilherme GASPARELLO, Otso TIRKKONEN, Sergio Luiz MOTA-JÚNIOR, Caroline Souza dos SANTOS, Felipe Calgaro MARTINS, Victoria Machado PEREIRA, Matheus Melo PITHON, Orlando Motohiro TANAKA

**Affiliations:** 1 Pontifical Catholic University of Paraná, School of Medicine and Life Sciences (Curitiba/PR, Brazil).; 2 Research Unit of Population Health University of Oulu (Oulu, Finland).; 3 The Wellbeing Services County of North Ostrobothnia (Oulu, Finland).; 4 Federal University of Rio de Janeiro, Dentistry Course (Rio de Janeiro/RJ, Brazil).; 5 State University of Southwest Bahia, Dentistry Course (Jequié/BA, Brazil).

**Keywords:** Orthodontics, Smile esthetics, Dental, Employability, Social perception, Ortodontia, Estética do sorriso, atratividade, Empregabilidade, Percepção social

## Abstract

**Objective:**

To evaluate the impact of orthodontic treatment on perceived smile attractiveness and hiring decisions in a Brazilian adult population.

**Methods:**

A cross-sectional observational study was conducted with 288 adult participants recruited via an online survey. Three clinical cases were selected, and standardized pre- and post-treatment smile photographs were shown in randomized order. Participants rated attractiveness on a 0-100 Visual Analog Scale (VAS) and indicated hiring intention (“yes,” “no,” or “can’t tell”). Descriptive statistics, Mann-Whitney U tests, chi-square tests, and logistic mixed-effects regression models were used for data analysis.

**Results:**

Post-treatment images showed significantly higher attractiveness ratings, compared with pre-treatment images (mean VAS: 54.2 vs. 36.4; p < 0.001). Hiring likelihood also increased after orthodontic treatment (44.0% vs. 27.9%; p < 0.001). Affirmative hiring decisions were consistently associated with higher attractiveness scores. In multivariable analysis, attractiveness was a significant independent predictor of hiring intention, while the interaction between VAS and treatment status was not significant (p = 0.201). Individual-level assessments demonstrated that many participants initially classified as non-hireable were reevaluated positively after treatment.

**Conclusions:**

Orthodontic treatment significantly improved smile attractiveness and increased hiring likelihood. Dental alignment alone was sufficient to shift social judgments, although overall facial esthetics also contributed. The identified attractiveness threshold marked the point at which orthodontic improvement began to influence employability perceptions. These findings highlight the psychosocial relevance of orthodontics beyond functional benefits.

## RESUMO

**Objetivo:**

Avaliar o impacto do tratamento ortodôntico na atratividade percebida do sorriso e nas decisões de contratação em uma população adulta brasileira.

**Métodos:**

Foi realizado um estudo observacional transversal com 288 participantes adultos recrutados por meio de uma pesquisa online. Três casos clínicos foram selecionados, e fotografias padronizadas do sorriso pré e pós-tratamento foram apresentadas em ordem aleatória. Os participantes avaliaram a atratividade em uma Escala Visual Analógica (EVA) de 0 a 100 e indicaram a intenção de contratação (“sim”, “não” ou “não sei”). Estatísticas descritivas, testes de Mann-Whitney U, testes qui-quadrado e modelos de regressão logística de efeitos mistos foram utilizados para análise dos dados.

**Resultados:**

As imagens pós-tratamento apresentaram avaliações de atratividade significativamente maiores, em comparação com as imagens pré-tratamento (média da EVA: 54,2 vs. 36,4; p < 0,001). A probabilidade de contratação também aumentou após o tratamento ortodôntico (44,0% vs. 27,9%; p < 0,001). Decisões afirmativas de contratação estiveram consistentemente associadas a escores de atratividade mais elevados. Na análise multivariada, a atratividade foi um preditor independente significativo da intenção de contratação, enquanto a interação entre EVA e status do tratamento não foi significativa (p = 0,201). Avaliações em nível individual demonstraram que muitos participantes inicialmente classificados como não contratáveis foram reavaliados positivamente após o tratamento.

**Conclusões:**

O tratamento ortodôntico melhorou significativamente a atratividade do sorriso e aumentou a probabilidade de contratação. O alinhamento dentário isoladamente foi suficiente para alterar julgamentos sociais, embora a estética facial geral também tenha contribuído. O limiar de atratividade identificado marcou o ponto em que a melhoria ortodôntica começou a influenciar as percepções de empregabilidade. Esses achados ressaltam a relevância psicossocial da Ortodontia além dos benefícios funcionais.

## INTRODUCTION

Dental appearance and smile esthetics play a fundamental role in facial attractiveness and social perception. Previous studies have demonstrated that dental and facial characteristics strongly influence judgments related to personality, intelligence, and professional competence.^1^ Orthodontic treatment, traditionally sought to improve occlusal function, is increasingly recognized for its psychosocial benefits, including improvements in self-esteem, quality of life, and perceived attractiveness.^2^ ^-^ ^4^

Aligned and harmonious smiles are consistently associated with higher ratings of facial attractiveness and favorable social judgments, whereas visible malocclusions, such as anterior open bite or dental crowding, may elicit negative evaluations.^5^ ^-^ ^7^ These perceptions extend beyond esthetics, influencing how individuals are viewed in professional and interpersonal contexts. For instance, research has indicated that dental esthetics can impact employability decisions, highlighting the weight of physical appearance in occupational opportunities.^8^ ^,^ ^9^

Experimental studies using pre- and post-orthodontic treatment images may help to evaluate the social consequences of orthodontic treatment. Controlled designs allow researchers to disentangle the role of dental alignment from other facial features, offering robust evidence of how orthodontic interventions shape social judgments.^5^ ^,^ ^10^

In Brazil, a country where dental esthetics are highly valued, exploring the intersection of orthodontics and social perception is particularly relevant. Malocclusions are not only clinical concerns but also social determinants of opportunities and well-being.^11^ ^,^ ^12^ Understanding these associations contributes to a broader discussion of how esthetic standards shape professional trajectories and reinforces the ethical imperative of considering psychosocial outcomes in orthodontic care.^1^ ^,^ ^12^

However, despite growing attention, gaps remain regarding how orthodontic treatment affects hiring perceptions across different sociocultural settings. For instance, several studies have reported that individuals are more likely to be considered for employment after orthodontic treatment.^8^ ^,^ ^9^ Nevertheless, it remains unclear to what extent orthodontic treatment alone drives these perceptions, and how much overall facial esthetics contribute to employability judgments, as previous studies have not isolated the specific impact of orthodontic treatment from the influence of overall facial appearance. Only a few investigations have attempted to address this distinction.

Therefore, the aim of this study was to evaluate the impact of orthodontic treatment on perceptions of smile attractiveness and employability judgments in a Brazilian population. Specifically, we investigated whether post-treatment smiles were rated as more attractive and increased the likelihood of being considered for hiring compared to pre-treatment images, as well as to identify the attractiveness threshold at which orthodontic treatment begins to significantly influence employability perceptions.

## METHODS

### STUDY DESIGN

This is a cross-sectional observational study assessing participants’ evaluations of hiring inquiries and visual attractiveness based on images presented. Ethical approval was not deemed necessary, as the study involved voluntary and anonymous participation without the collection of identifiable information. All participants provided informed consent, and the individuals whose smile images were utilized also granted explicit permission for their use in research and educational contexts. This study was conducted in full accordance with the principles outlined in the Declaration of Helsinki and its subsequent revisions, ensuring ethical rigor in all procedures.

### ORTHODONTIC TREATMENT

Three patients were selected from a record of a private orthodontic clinic. This process was conducted by a panel of three orthodontic specialists, who collaboratively selected three representative cases illustrating typical clinical scenarios: 1) a patient with an anterior open bite, 2) a patient presenting with facial features associated with dental crowding, and 3) a patient who underwent orthognathic surgery as part of a surgery-first approach. 

### IMAGE PREPARATION

The images were captured before and after orthodontic treatment. The images were captured with a Canon EOS Rebel T7 DSLR camera and a 100 mm macro lens against a plain white background. The camera was positioned 1.5 meters away at the patient’s mouth level.

### SAMPLE SIZE CALCULATION

The sample size was calculated using the Brazilian population as a reference, under the assumption of an infinite population. A 95% confidence level and a 6% margin of error were applied, and the standard formula for estimating proportions was used to determine the minimum required sample size, which was calculated to be 267 participants. A total of 288 participants aged 18 years or older responded, exceeding the minimum requirements.

## DATA COLLECTION

Data were collected via the Qualtrics online survey platform Qualtrics™, Provo, Utah, USA from April 26 to May 8, 2025, targeting residents of Brazil. Participants were shown three pretreatment smile images and three respective post-treatment images. The presentation order of the images within each set was randomized to minimize potential order effects and response bias. All responses were submitted electronically during this period. 

While spectating each image, participants answered following inquiries:

» Hiring task

*“Would you consider hiring this person”*

Participants responses were recorded with categorical choice of “yes” / “no” / “can’t tell from the image”.

» Attractiveness rating

*“On a scale from 0 to 100, how attractive do you consider this smile?”*

0 = Not attractive at all; 100 = Extremely attractive.

In addition to evaluating the images, participants were asked to complete a brief demographic questionnaire to help describe the study population. The information collected included age, sex, employment status, marital status, and region of residence within Brazil.

### PILOT STUDY

A pilot study with 40 participants mean age = 32.6 years, SD = 8.4 was conducted to assess the reliability of the questionnaire. Each participant completed the same questionnaire twice, 20 days apart. The results indicated excellent reliability for the Visual Analog Scale (VAS). Intraclass Correlation Coefficient (ICC) was 0.83, and no significant differences were found between the two time points (paired t-test for VAS: t = 0.61, p = 0.613). Responses from the pilot test were excluded from the main analysis to ensure participant anonymity.

### STATISTICAL ANALYSIS

Survey responses were exported from Qualtrics and analyzed using R software version 4.4.0.^13^ Descriptive statistics including frequencies, means, and standard deviations were computed. The Mann-Whitney U test was utilized to compare VAS scores before and after orthodontic treatment. Hiring responses were examined using the chi-square test. Boxplots were created to visualize hiring response distributions. A logistic mixed-effects regression model assessed the relationship between VAS scores and binary hiring decisions. This model incorporated a random intercept for each participant to account for repeated measures and tested the interaction between VAS and orthodontic treatment status. Predicted probabilities of hiring decisions with 95% CI were plotted and predicted hiring probability thresholds of 0.5 was calculated. Additionally, we fitted a generalized linear mixed model GLMM with a binomial logit link to test whether the relationship of VAS ratings and hiring decisions differed between before and after orthodontic treatments. The model included a random intercept for the participant due to the repeated measures. Eventually, individual trajectories of hiring status before and after orthodontic treatment were plotted. All visualizations were produced using the “ggplot2” and “MASS” packages.^14^ ^,^ ^15^

## RESULTS

Participants came from various regions across Brazil, with the majority 62.9%, n = 180 residing in the Southern region (Table 1). The average age was 32.4 years, and 63.4% identified as female.

**Table 1:** Characteristics of study participants.

Variable name	% (n)
Education	
High School Diploma or Equivalent	34.7 (100)
Undergraduate Degree	25.7 (74)
Postgraduate Degree (Specialization)	20.8 (60)
Postgraduate Degree (Master's or Doctorate)	18.8 (54)
Not Reported	0
Sex	
Male	36.6 (105)
Female	63.4 (182)
Not reported	1
Employment	
Retired	1.39 (4)
Self-employed or Entrepreneur	28.5 (82)
Unemployed or Temporarily Laid Off	2.08 (6)
Employed in a Permanent / Full-Time Position	29.9 (86)
Full-Time Student	28.5 (82)
Full-Time Student and Part-Time Employee	6.60 (19)
Other	3.13 (9)
Not Reported	0
Marital status	
Married	38.5 (111)
Divorced or Separated	4.17 (12)
Single	56.9 (164)
Widowed	0.347 (1)
Not Reported	0
Region	
Central-West	9.44 (27)
Northeast	10.8 (31)
North	1.40 (4)
Southeast	15.4 (44)
South	62.9 (180)
Not Reported	2
Age [mean (SD)]	32.4 (12.0)
Not Reported	3

Participants preferred post-orthodontic treatment images compared to the pre-orthodontic treatment images (VAS 36.4 vs. 54.2; p-value < 0.001). Likewise, participants were more likely to consider hiring a person after orthodontic treatment compared to pretreatment situations (44.0% vs. 27.9%). The difference in hiring decisions was statistically significant (p-value < 0.001) (Table 2).

**Table 2:** VAS evaluations and hiring decisions stratified by orthodontic treatment status (before vs. after).

	Before treatment	After treatment	p-value
	Mean (SD)	Mean (SD)	
VAS score	36.4 (26.3)	54.2 (27.6)	< 0.001^a^
Missing	160	147	
Would consider to hire this person	% (n)	% (n)	
Yes	27.9% (213)	44.0% (327)	< 0.001^b^
No	24.9% (190)	11.9% (89)	
Can’t tell	47.2% (361)	44.1% (328)	
Missing	100	120	

^a^ Tested Man Whitney U test, ^b^ Tested with chi-square test.

**Figure 1:**
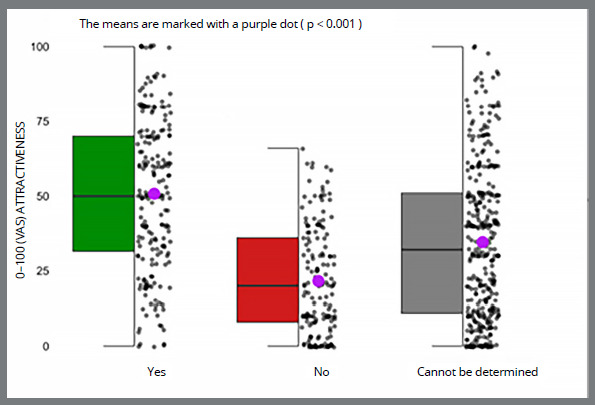
VAS attractiveness scores according to hiring decision prior to orthodontic treatment.

The distributions of affirmative hiring before orthodontic treatment were distributed equally across the VAS scale, with a mean of 50.8 (Fig. 1). Negative responses were more skewed towards lower VAS ratings, with a mean of 21.6. VAS evaluations for participants hired were significantly better for affirmative hiring decisions compared to the negative ones p < 0.001.

**Figure 2:**
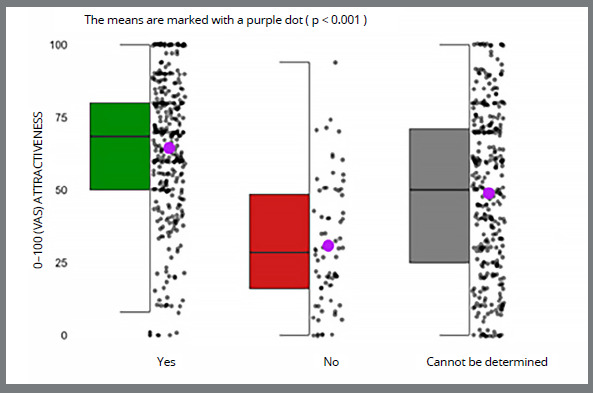
VAS attractiveness scores according to hiring decision after orthodontic treatment

After orthodontic treatment, only a few participants with VAS scores in the lowest quartile had affirmative hiring decisions, with a mean of yes answers of 43.2 (Fig. 2). Likewise, in pre-orthodontic treatment images, participants evaluated superior VAS scores for participants with affirmative hiring decisions compared to negative ones 43.2 vs. 23.4; p-value < 0.001.

**Figure 3:**
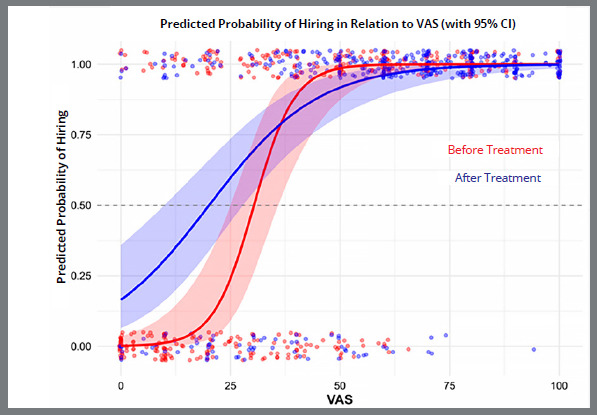
Predicted probability of hiring as a function of VAS attractiveness ratings before (red) and after (blue) orthodontic treatment.

Average VAS for pre-orthodontic treatment images hiring probability of 0.5 was calculated to be 30.4, whereas the respective VAS score for post-orthodontic treatment hiring probability was 20.1 (Fig. 3). The interaction between VAS ratings and orthodontic treatment Before vs. After was not statistically significant p = 0.201, indicating that the evaluation of VAS did not differ significantly between before and after orthodontic treatment in hiring decisions. Both before and after orthodontic treatment images with the highest VAS scores were the most likely to be selected for hiring, whereas images with lower VAS scores had a considerably lower probability of being chosen.

**Figure 4:**
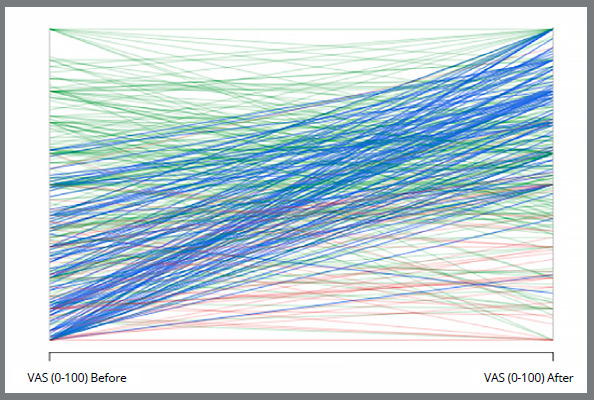
Individual trajectories of hiring status before and after orthodontic treatment. Blue lines represent participants who became hireable after treatment, green lines those consistently considered hireable, and red lines those who were not considered hireable at either time point.

The visualization of individual trajectories highlights the strong influence of orthodontic treatment on hiring evaluations and VAS ratings (Fig. 4). The predominant blue lines represent participants who were not considered for hiring before treatment but were positively evaluated afterwards, reflecting a substantial gain in employability. Importantly, these participants also demonstrated a clear increase in VAS scores following treatment, reinforcing the association between improved dental esthetics and hiring decisions. Green lines indicate participants consistently hired at both time points, suggesting that orthodontic treatment helped sustain already favorable evaluations. By contrast, red lines, representing a small minority, correspond to individuals who did not get hired regardless of orthodontic treatment at any moment. Overall, the predominance of blue trajectories, coupled with their marked improvement in VAS scores, underscores that orthodontic treatment significantly enhanced both perceived attractiveness and the likelihood of being hired.

## DISCUSSION

The present study demonstrated that orthodontic treatment positively influenced both perceptions of smile attractiveness and employability judgments. Participants consistently rated post-treatment images as more attractive and were more likely to indicate willingness to hire compared to pretreatment conditions. These findings align with previous reports that malocclusions negatively affect social judgments, whereas esthetically improved smiles enhance perceptions of competence, intelligence, and social acceptance.^8^ ^,^ ^9^ ^,^ ^16^ ^,^ ^17^

The present findings demonstrate that attractiveness ratings significantly shaped hiring decisions, reinforcing prior evidence that dental alignment functions as a strong visual cue in social and professional evaluations. Before orthodontic treatment, affirmative hiring decisions were associated with higher VAS ratings (mean = 50.8), compared to negative ones (mean = 21.6), reflecting patterns reported by a previous study^5^, which showed that both laypersons and professionals are sensitive to variations in dental esthetics. Similarly studies found that aligned teeth contribute to improved self-perception and oral health-related quality of life, which are mirrored here in the more favorable employability judgments for candidates with higher attractiveness ratings.^4^ ^,^ ^13^ These results confirm that dental esthetics are not merely superficial but carry measurable consequences for social valuation and occupational opportunities. 

Following orthodontic treatment, the probability of being hired remained strongly linked to attractiveness, with candidates in the highest VAS quartile almost certainly hired, while those in the lowest quartile remained disadvantaged. Although the interaction between VAS ratings, treatment, and hiring decisions was not statistically significant p = 0.201, treatment was associated with a shift of approximately 10 points on the VAS scale. Even though this difference did not reach statistical significance, it suggests that treatment may move marginal cases closer toward affirmative hiring evaluations, corroborating previous studies^18^ ^,^ ^19^ This study highlights the transformative effect of orthodontic treatment on employability. While previous research largely documented the negative consequences of poor dental appearance on employment prospects;^8^ ^,^ ^9^ ^,^ ^20^ ^,^ ^21^ few studies have shown clear evidence of benefits after treatment;^22^ ^,^ ^23^ Our findings provide support for such benefits, demonstrating that orthodontic treatment substantially improved hiring evaluations and VAS ratings. Most participants who were initially overlooked were positively reassessed after treatment, indicating that enhanced dental esthetics not only improved perceived attractiveness but also translated into greater employability.

In addition, orthodontic treatment has been associated with functional and quality-of-life benefits that indirectly enhance social perception. Improvements in oral function, speech, and mastication have been reported, further contributing to overall well-being.^3^ ^,^ ^24^ Studies also demonstrate reductions in bullying and peer victimization following orthodontic treatment, which underscores the importance of dental esthetics in social integration.^25^ ^-^ ^27^ From an occupational standpoint, esthetic improvements contribute to a more professional appearance, which recruiters may interpret as reflecting responsibility and competence.^7^ ^,^ ^8^ Collectively, these findings reinforce that orthodontics extends beyond occlusal correction, functioning as a determinant of psychosocial health and employability. 

This study is not without limitations. First, the cross-sectional design limits causal inferences regarding the relationship between orthodontic treatment and employability judgments. Second, the study was restricted to Brazilian participants, which may limit generalizability to other cultural contexts, as perceptions of esthetics vary internationally.^27^ ^,^ ^28^ Moreover, the use of only three images in a two-dimensional shape, while controlled, does not fully replicate real-world judgments where dynamic facial cues and personality traits also play significant roles.^5^

Future studies should include longitudinal designs, larger and more diverse populations, and experimental manipulations that isolate dental alignment from other facial esthetic features. Although, our findings support that orthodontic treatment significantly enhances both smile attractiveness and perceived employability. Aligned teeth alone are sufficient to shift judgments positively, yet the overall improvement in facial esthetics may further amplify these perceptions. Beyond cosmetic benefits, orthodontics contributes to self-esteem, psychosocial well-being, and social integration, which collectively improve professional opportunities. While limitations exist, the present results reinforce the broader social value of orthodontic care. 

## CONCLUSION

Orthodontic treatment significantly improved smile attractiveness and increased the likelihood of being considered for hiring. Aligned teeth by themselves were sufficient to positively shift social judgments, although overall facial esthetics also appeared to play a role. The identified turning point in attractiveness ratings demonstrated where orthodontics begins to influence employability perceptions. These findings highlight the broader psychosocial relevance of orthodontics beyond functional outcomes.

## Data Availability

Due to the sensitive nature of the data and ethical restrictions, the datasets supporting this study are not publicly available.
